# Vitamin D Supplementation and Cancer Mortality: Narrative Review of Observational Studies and Clinical Trials

**DOI:** 10.3390/nu13093285

**Published:** 2021-09-21

**Authors:** Patrizia Gnagnarella, Valeria Muzio, Saverio Caini, Sara Raimondi, Chiara Martinoli, Susanna Chiocca, Claudia Miccolo, Paolo Bossi, Diego Cortinovis, Ferdinando Chiaradonna, Roberta Palorini, Federica Facciotti, Federica Bellerba, Stefania Canova, Sara Gandini

**Affiliations:** 1 Division of Epidemiology and Biostatistics, IEO European Institute of Oncology IRCCS, 20141 Milan, Italy; valeria.muzio@studenti.unimi.it; 2 Institute for Cancer Research, Prevention and Clinical Network (ISPRO), Via Cosimo il Vecchio 2, 50139 Florence, Italy; s.caini@ispro.toscana.it; 3 Department of Experimental Oncology, IEO, European Institute of Oncology IRCCS, 20141 Milan, Italy; sara.raimondi@ieo.it (S.R.); Chiara.Martinoli@ieo.it (C.M.); susanna.chiocca@ieo.it (S.C.); claudia.miccolo@ieo.it (C.M.); Federica.Facciotti@ieo.it (F.F.); Federica.Bellerba@ieo.it (F.B.); sara.gandini@ieo.it (S.G.); 4 Medical Oncology, Department of Medical and Surgical Specialties, Radiological Sciences and Public Health University of Brescia, ASST-Spedali Civili, 25121 Brescia, Italy; paolo.bossi@unibs.it; 5 SC Oncologia Medica, Asst H S Gerardo Monza, 20900 Monza, Italy; d.cortinovis@asst-monza.it (D.C.); s.canova@asst-monza.it (S.C.); 6 Department of Biotechnology and Biosciences, University of Milano-Bicocca, 20126 Milan, Italy; ferdinando.chiaradonna@unimib.it (F.C.); roberta.palorini@unimib.it (R.P.)

**Keywords:** cancer, vitamin D, supplementation, mortality, survival

## Abstract

Several studies have investigated the beneficial effects of vitamin D on survival of cancer patients. Overall evidence has been accumulating with contrasting results. This paper aims at narratively reviewing the existing articles examining the link between vitamin D supplementation and cancer mortality. We performed two distinct searches to identify observational (ObS) studies and randomized clinical trials (RCTs) of vitamin D supplementation (VDS) in cancer patients and cohorts of general population, which included cancer mortality as an outcome. Published reports were gathered until March 2021. We identified 25 papers published between 2003 and 2020, including n. 8 RCTs on cancer patients, n. 8 population RCTs and n. 9 ObS studies. There was some evidence that the use of VDS in cancer patients could improve cancer survival, but no significant effect was found in population RCTs. Some ObS studies reported evidence that VDS was associated with a longer survival among cancer patients, and only one study found an opposite effect. The findings do not allow conclusive answers. VDS may have the potential as treatment to improve survival in cancer patients, but further investigations are warranted. We strongly support investment in well-designed and sufficiently powered RCTs to fully evaluate this association.

## 1. Introduction

Vitamin D is highly important for bone health and mineral metabolism, but more recently interest has been driven by its non-musculoskeletal functions. Vitamin D deficiency is linked with numerous illnesses, including osteoporosis and osteomalacia, autoimmune disorders, infectious diseases, muscle weakness and falls, cardiovascular diseases (CVDs), cancers, and neurological disorders [1]. The vitamin D status in the body is mainly dependent on the exposure to ultraviolet (UV) light. It is well established that the main source of vitamin D3 occurs via skin exposure to UV light from the sun rather than by food intake. Vitamin D production is initiated in the skin by UVB with the conversion of 7-dehydrocholesterol into pre-vitamin D3 in the skin, followed by two successive hydroxylations in the liver, to produce 25-hydroxycholecalciferol [25(OH)D] and then in the kidneys to produce the active metabolite, 1,25-dihydroxycholecalciferol [1,25(OH)2D]. Dietary intake of vitamin D, mainly from fish oils, fish, egg yolks, and to a lesser extent mushrooms, and from dietary supplements, accounts for a small amount of the daily vitamin D requirement. Inadequate exposure to sunlight and dietary intake of vitamin D may affect cancer incidence and mortality [2,3].

Evidence from observational studies indicates that low vitamin D status is associated with higher mortality for cancer and cardiovascular disease [4,5]. Several epidemiological studies, including both ObS studies and RCTs, have investigated the alleged beneficial effects of vitamin D on survival and mortality of patients with cancer. Vitamin D acts as an effective regulator of cell growth and differentiation in several different cell types, including cancer cells [6]. Findings from ObS studies constitute evidence suggestive of a relationship between Vitamin D and cancer survival and mortality, but they are insufficient to establish causality. A recent critical appraisal of meta-analyses found that vitamin D supplementation did not affect cancer incidence, but a weak reduced total cancer mortality risk emerged from the analyses’ results. Specifically, five out of six meta-analyses reported a risk reduction up to 16%, due to trials with small sample size and follow-up and not adequately powered to detect cancer outcomes [7].

Numerous trials assessing the effect of vitamin D supplementation on different outcomes are currently available. Among these, the VITAL, RECORD and ViDA trials are the largest in terms of number of participants. The VITAL (Vitamin D and Omega 3 Trial) randomized more than 25,000 participants for the prevention of cancer and cardiovascular disease among men 50 years of age or older, and women 55 years of age or older in the United States [8]. The RECORD Trial recruited more than 5000 participants aged at least 70 years reporting fragility fracture within the last 10 years from 21 orthopedic centers in the United Kingdom (https://www.thelancet.com/protocol-reviews/02PRT-35 accessed on 19 July 2021). Participants were randomly allocated to daily vitamin D3 (800 IU), calcium (1000 mg), both or placebo for 24–62 months, with a follow-up of 3 years after intervention. The Vitamin D Assessment (ViDA) study was a RCT carried out in New Zealand to examine whether high-dose vitamin D supplementation received monthly, without calcium, was associated with a reduction in cancer incidence and cancer mortality in community adults (in post hoc analysis). None of these trials confirmed the benefit of vitamin D3 supplementation on overall mortality [9,10,11].

Our group previously examined the mortality in subjects who participated in RCTs, testing the impact of vitamin D supplementation (i.e., vitamin D2 or vitamin D3) on any health condition [12]. This meta-analysis pooled 18 trials among various study populations vitamin D concentrations, followed for a mean 5.7 years. We found that ordinary doses of vitamin D supplements (300 to 2000 IU) were associated with a significant decrease in total mortality rates and no between-study heterogeneity.

The past two decades have witnessed a vigorous increase in interest in vitamin D from both the lay and biomedical worlds. Much of the growing interest is powered by new data available and the interest in repurposing drugs as anticancer therapeutics that could shorten the conventional investigational pathway and open multiple new avenues of investigation [13]. Because of the conflicting evidence, limitations of previous reviews and availability of new data, we here present a narrative review of RCTs and ObS studies examining the impact of vitamin D supplementation on cancer mortality.

## 2. Materials and Methods

A systematic literature search was conducted. We performed two distinct searches to identify RCTs and ObS studies. RCTs were proposed in cancer patients or in cohorts of general population in which vitamin D supplementation was provided and cancer mortality was reported as a trial outcome. We searched for ObS studies with different study designs investigating the association between previous vitamin D supplementation and reporting cancer mortality estimates.

Published reports were gathered from the following databases: PUBMED, EMBASE and ISI Web of Knowledge up until March 2021.

We searched the following MeSH terms and keywords: “supplementation”, “Vitamin D” or “cholecalciferol”, “RCT” or “epidemiologic studies” or “cohort”, “cancer”, “neoplasm” or “tumor”, and “mortality”, “survival” and “outcome”, without any restriction. We also performed manual search of references cited in the retrieved articles and preceding reviews on the topic.

Titles and abstracts were screened by two researchers (P.G. and V.M.), who then assessed full texts for eligibility.

The inclusion criteria were based on the PICO’S framework [14]. Regarding participants, we considered all the individuals over the age of 18 years. All the selected studies present an intervention with vitamin D supplementation or a reported past use of vitamin D supplementation. The supplemented groups were compared with those treated with a placebo, a lower dose of vitamin D or no use of vitamin D supplementation. The main outcome was cancer mortality or all measures of survival, progression-free survival (PFS) and overall survival (OS) in cancer patients. According to the study design, both RTC and ObS were included.

### Data Extraction

A standardized data-collection protocol was used to gather the relevant data from each selected article. The data from eligible studies were extracted into a designed database, including the following information from each publication: authors, journal and year of publication, country, study population, type of study and cohort/trial name, sample size, sex distribution, mean age and standard deviation (SD) or range, inclusion criteria, primary and secondary outcomes. Concerning vitamin D supplementation, we recorded number of study arms, type of supplementation, daily or weekly dose and duration of use, comparators and time of follow-up. When available, we reported the measured outcome fully adjusted (e.g., Hazard Ratio (HR) for OS, disease free-survival (DFS) or Relative Risk (RR)). Due to the relevance of possible confounders in the studied association, only adjusted estimates were considered. We considered the placebo arm group as reference group in our analyses for RCT and vitamin D, no users in ObS studies. Because of the heterogeneity of studies and the lack of appropriate control of major confounding factors, we decided that it was not appropriate to perform a meta-analysis of their results.

Articles were reviewed and data were extracted and crosschecked independently by two investigators (V.M. and P.G.). Any disagreement at any stage was resolved by consensus among the two or within the working group.

## 3. Results

**Figure 1:**
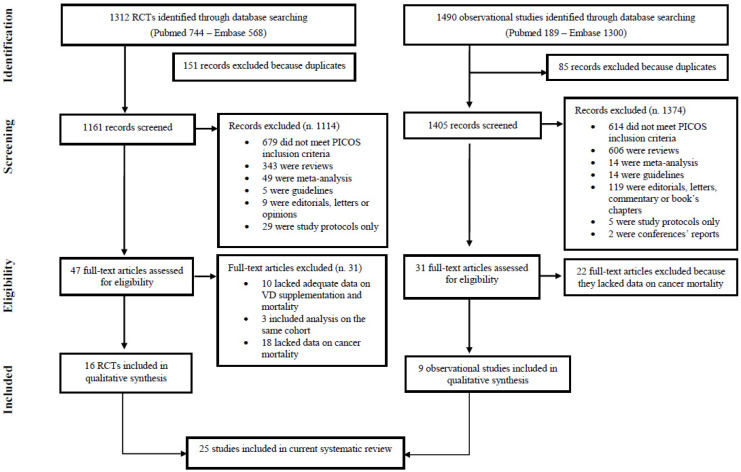
Flowchart of study selection.

The literature search yielded a total of 1312 RCTs and 1490 ObS studies, according to the two searching strategies. Figure 1 represents the double flowchart of the selection process. Regarding the RCTs, after eliminating the duplicates, we found 1161 publications, of which 47 were assessed for eligibility. Upon subsequent review of the full-text articles identified, we excluded further 31 studies either because they lacked adequate data on the association between vitamin D supplementation and mortality, were not independent, or in which data on cancer mortality were missing. As a result, this review covers 16 RCTs. We performed the same selection process for observational studies. Likewise, after eliminating the duplicates, we collected 1405 records, of which 31 were assessed for eligibility. The assessment of the full text articles led us to exclude additional 22 studies which lacked data on cancer mortality. Finally, nine ObS studies were included in the review.

### General Characteristics of Studies

The main characteristics of the included studies are summarized in Table 1a–c. Among the 16 identified RCTs, eight were carried out on cancer patients [15,16,17,18,19,20,21,22] (Table 1a) and eight were population trials [9,10,11,23,24,25,26,27] (Table 1b); as previously reported, the ObS studies were 9 [28,29,30,31,32,33,34,35] (Table 1c).

Regarding RCTs on cancer patients, we included three publications on prostate, two of which were on the Androgen-Independent Prostate Cancer Study of Calcitriol Enhancing Taxotere (ASCENT) study [15,17], and two on digestive tract cancers (from esophagus to rectum), both of the AMATERASU trial, a randomized, double-blind, placebo-controlled trial conducted in Japan [21,22]. Two trials were conducted on metastatic colorectal cancer (CRC) [19,20], and one study on Non-Small-Cell Lung Cancer [18]. Altogether, four trials were conducted in North America [15,16,17,20], two in Japan [21,22], and one in Croatia [19], while publication year ranged from 2007 to 2019. In total, 1806 cancer survivors were randomly assigned to dietary supplements or placebo, ranging from 24 to 92 years of age (Table 1a).

Most of the population trials were conducted in the United States (five studies) [10,24,25,26,27], two were conducted in the United Kingdom [9,23], and only one in New Zealand [11]. We included four reports on the Women’s Health Initiative, which had the largest size (36,282 participants) among all included trials. This is a randomized controlled trial of Calcium/Vitamin D supplementation compared with placebo in postmenopausal women [24,25,26,27]. The remaining trials enrolled individuals of both sexes and the total sample size was 75,239 persons, ranging from 50 to 84 years of age (Table 1b).

Out of nine ObS studies included, two were cohort studies [28,29], one had a retrospective study design [30], one was a case–control study [31], one was a longitudinal study [32], three used data from the National/Local Cancer Registry [33,34,36] and one reported data of a consortium of four prospective cohorts from the United States and China including 12,000 breast cancer patients [35] (Table 1c). All studies were published between 2013 to 2018. The total sample size was 41,971 cancer survivors, ranging from 18 to 89 years of age at cancer diagnosis. Five studies included only women [28,30,33,34,35] and four both sexes [29,31,32,36]. The average follow-up time ranged from 1.8 to 13.1 years. In Table 1a–c are reported the inclusion criteria and primary and secondary outcomes.

Table 2a–c summarizes the main characteristics of the selected studies and the HR with corresponding 95% CI, adjusted for the maximum number of confounding variables for OS, cancer mortality, and cancer-specific mortality.

Regarding the RCTs on cancer patients, all the included studies were two-arms [15,16,17,18,19,20,21,22]. Four studies were placebo-controlled trials [18,19,21,22] consisting of a prescription of vitamin D, in doses of UI (International Units) compared to placebo. Only in the SUNSHINE trial [20] did the control group receive a standard dose of vitamin D (400 UI/day). The supplementation ranged from a minimum of 1200 UI to a maximum of 8000 UI, with a single dose a day. In two studies, the vitamin D supplementation was provided in association with prescribed therapy. In the ASCENT study, they administered 45 mcg of DN-101 (high dose of calcitriol) in association with 36 mg/m^2^ docetaxel, and 24 mg dexamethasone weekly for 3 weeks of a 4-week cycle [15,17], and in Attia [16] they administered a 4-week cycle of docetaxel (35 mg/m^2^ i.v., days 1, 8, and 15) with or without doxercalciferol (10 mcg orally, days 1–28) (Table 2a).

Among population RCTs, seven were two-arm trials [10,11,23,24,25,26,27] and only one was a four-arm trial [9]. Considering vitamin D supplementation, the intervention varied among the studies. In almost all the studies, the intervention was characterized by a daily dose of vitamin D with the exception of VIDA trial, in which a first load of 200,000 UI was followed by a monthly supplementation of 100,000 UI [11] (Table 2b).

In two of the analyzed cohorts, WHI and RECORD trial, the Vitamin D supplementation was associated with 1000 mg of calcium [9,24,25,26,27]. In the VITAL trial, 2000 UI of vitamin D were supplemented with 1 g of omega 3 fatty acids [10]. All the RCTs on population followed the intervention from a minimum of 3 years to a maximum of 7 years. None of the included studies reported a statistically significant effect of VDS compared to placebo group in term of overall cancer mortality or specific-cancer mortality.

In the included studies, OS was expressed as HR and in some cases as median in months [16,17,20] comparing VDS versus placebo. VDS was associated with a significant increased OS rate in three studies [15,20,22]. The strongest effect was found in the AMATERASU trial on cancer digestive tract [20]. In a post hoc analysis among patients with poorly differentiated adenocarcinoma, they found a 5-year OS rate of 92% in the vitamin D group compared with 72% in the placebo group (HR = 0.25; 95% CI, 0.07–0.94; P = 0.04). Considering the median OS expressed in months, Attia [16], Ng [20] and Scher [17] reported no significant difference between the treatment and the placebo or standard vitamin D dose groups (Table 2c).

Regarding observational studies, the included populations were stratified for different levels of supplementation, or for past vitamin D use. Jeffreys and colleagues analyzed the pre-diagnostic different VDS (three or more versus one to two prescriptions of vitamin D and any vitamin D prescription compared to no past use of vitamin D supplements) in a group of women with a first diagnosis of breast, CRC, lung, ovarian or uterine cancer between 2002 and 2009 [33]. Yokosawa considered three different levels of vitamin D supplementation (from 0 UI to 400 UI and >400 UI/die) [29]. The follow up ranged from 1.3 weeks to 7.0 ± 1.4 years (Table 2c).

The included studies reported the OS rate expressed as HR or cancer-specific survival rate. Four studies reported a statistically significant improved survival [30,31,33,34]. Mulpur et al. found that the use of vitamin D in GBM patients was associated with a reduced mortality, adjusting for age and other covariates (HR = 0.72; 95% CI 0.52–0.99) [31]. The remaining studies found a beneficial effect of VDS in breast cancer patients. Zeichner [30] found that VDS use was associated with an improved OS (HR, 0.31; 95% CI, 0.11–0.89) in women who received VD supplementation during neoadjuvant chemotherapy. Madden [34] found a 20% reduction in breast cancer-specific mortality in de novo vitamin D users compared to non-users (HR 0.80; 95% CI 0.64–0.99), analyzing records of invasive breast cancer patients identified on the National Cancer Registry Ireland database. The reduction was greater (HR 0.51; 95% CI 0.34–0.74) when vitamin D was initiated after the breast cancer diagnosis (within 6 months). Jeffreys [33] found that exposure to three or more versus one to two prescriptions of VD was not associated with survival from any of the four cancers studied (CRC, lung, gynecological, breast), but they found that any VD prescription, compared with no past prescriptions, was associated with a better survival from breast cancer (HR 0.78, 95% CI 0.70–0.88). Only Holm [28] reported a higher BC specific mortality (HR: 1.47; 95% CI, 1.07–2.00) in women with high pre-diagnostic intake of vitamin D supplements associated with hormone replacement therapy (HRT) (Table 2c).

## 4. Discussion

We conducted a narrative review to extensively address the effect of VDS use on overall cancer mortality or cancer-specific mortality, in RCTs conducted among cancer patients or the general population and in observational studies. This is the first narrative review to simultaneously and critically evaluate the scientific evidence of the effect of Vitamin D supplementation on cancer survival and mortality. Because of the heterogeneity of studies, we decided that it was not appropriate to perform a meta-analysis of their results. We identified 25 papers published between 2003 and 2020. There was limited evidence that the use of VDS could reduce cancer-related mortality among cancer patients, but no effect on mortality was found in population trials. Some observational studies reported evidence that VDS was associated with a longer survival among cancer patients, and only one study [28] found an opposite effect.

The reason for the divergent findings for cancer mortality is not clear. There are plausible mechanisms for the operation of vitamin D in decreasing tumor invasiveness and propensity to metastasize, and influencing immunomodulatory properties [37] that may contribute to reduced metastatic disease and fatal cancer [2]. Vitamin D deficiency prevalence is high in cancer patients [38,39,40] and some studies report vitamin D deficiency in more than 70% of cancer patients. According to Alkan, risk factors linked with vitamin D deficiency include female sex, low sunlight exposure, being under palliative care or adjuvant chemotherapy or history of gastrointestinal surgery [40]. Moreover, the VDS dose may have been inadequate to sufficiently increase vitamin D levels. In the AMATERASU trial, Urashima found the 5-year relapse-free survival was higher than placebo (85% vs. 71%) in the subgroup of patients with 25(OH)D between 20 and 40 ng/mL, but not in patients with level <20 ng/mL [21]. Meanwhile, Ng found a greater effect of high-dose vitamin D3 (8000 IU VD3/day followed by 4000 IU VD3/day during chemotherapy) on PFS among patients with a lower BMI (*P* = 0.04 for interaction), more metastatic sites (*P* = 0.02 for interaction), and KRAS wild-type tumors (*P* = 0.04 for interaction) in the SUNSHINE trial [20]. In a recent meta-analysis of Vitamin D supplementation in RCTs [41], authors found a statistically significantly reduced 15% risk of cancer death (RR = 0.85,CI = 0.74–0.97) and subgroup analyses suggest that all-cause mortality may be significantly lower in trials with vitamin D3 supplementation than in trials with vitamin D2 supplementation (P for interaction = 0.04) [41]. In fact, Vitamin D3 seems to be more efficient at raising serum 25(OH)D concentrations than vitamin D2, and thus vitamin D3 could potentially become the preferred choice for supplementation [42].

The need for a long follow-up period is necessary to evaluate the possible effect of VDS. Most of the included trials on cancer patients had follow-up periods of no more than 4 years [19], while population trials have a longer follow-up, up to 7 years. Among the largest of these trials is the Women’s Health Initiative (WHI). The WHI randomized >36,000 US postmenopausal women to 7 years of daily calcium and vitamin D3 or to placebo and found a suggestion of a protective effect against overall cancer mortality [26], CRC [24], breast [25] and hematologic cancer mortality [27], but the dose was below the Recommended Dietary Allowance (RDA, 600 UI) (https://ods.od.nih.gov/factsheets/VitaminD-HealthProfessional/ accessed on 19 July 2021) and the VDS proposed in the other trials.

A recent systematic overview of pertinent meta-analyses [7] found that VDS reduced total cancer mortality risk in population clinical trials, with five out of six meta-analyses reporting a relative risk (RR) reduction of up to 16% (RR 0.84, 95% CI = 0.74–0.95). We did not perform a meta-analysis of the included studies due to the great heterogeneity in reported estimates. Most published trials did not present adjusted estimates for general or specific cancer mortality, but they all showed a promising indication of reduction in total cancer mortality. In VITAL, VDS did not significantly reduce total cancer mortality (HR = 0.83, 95% CI = 0.67–1.02), but accounting for latency by excluding the first year or first two years of follow-up, they found a statistically significant cancer mortality reduction (HR = 0.79; 95% CI = 0.63–0.99 and HR = 0.75; 95% CI = 0.59–0.96 respectively).

Regarding RCT on cancer patients, two trials (ASCENT and SUNSHINE) evaluated the effect of VDS in patients with progressive metastatic prostate cancer and metastatic CRC, respectively. The ASCENT interventions proposed different VDS [15]. They evaluated the safety and activity of DN-101, a new high-dose oral formulation of calcitriol designed for cancer therapy, and docetaxel compared with placebo and docetaxel (chemotherapy). They found a trend favoring DN-101 over placebo with regard to skeletal morbidity-free survival. According to the authors, the observed trends could reflect a more effective anti-neoplastic therapy (with DN-101) resulting in delay of skeletal-related events (bone metastases). The SUNSHINE trial provided mFOLFOX6 plus bevacizumab chemotherapy every 2 weeks and either high-dose vitamin D3 (8000–4000 UI/day) or standard-dose vitamin D3 (400 UI/day) daily until disease progression or intolerable toxicity. Several potential mechanisms of action may explain the activity of vitamin D in CRC, but the hypothesis-generating finding could be that high-dose vitamin D3 supplementation is responsible in maintenance of gut mucosal barrier integrity [43]

In the AMATERASU trial, the authors found a significant effect of VDS in the subgroup of patients with digestive tract cancer with poorly differentiated adenocarcinoma, but not in any other subgroup based on histopathological characteristics [22]. The authors concluded that VDS could induce/increase differentiation of undifferentiated cancer cells as reported in in vitro experiments [44,45,46] and in a clinical pilot trial [47].

Regarding observational studies, four studies have reported a statistically significant improved survival [30,31,33,34], and only one study [28] found a contrasting result. The “Danish Diet, Cancer and Health” found that the use of VDS was significantly associated with a 47% increase in breast cancer mortality in women reporting a pre-diagnostic use of HRT. This association could be due to the fact that previous HRT users could carry the worst prognosis compared to both never and current users as reported in WHI trial (more advanced tumors and higher frequency of lymph node positive tumors) [48].

Madden found that de novo post-diagnostic VDS was associated with a 20% reduction in breast cancer-specific mortality in a large cohort study [34]. In additional analysis, they also found a greater reduction in breast cancer-specific mortality (49%) when VDS was initiated soon after the breast cancer diagnosis (within 6 months), similar to Jeffreys [33], who examined over 11,000 breast cancer patients from the largest prospective cancer cohort included in this review. They found that any vitamin D prescription, compared to never receiving it, in the 5 years prior to diagnosis was associated with improved survival from breast cancer, but not for the other cancer types (CRC, lung, ovarian and uterine). Time of initiation of VDS could have significant clinical implications, particularly for breast cancer patients, but further studies are needed to clarify the association.

Moreover, another aspect to be considered is vitamin D receptor polymorphism, which could explain the different activity of VDS. There are several reports linking vitamin D receptor genotype with cancer risk and mortality, which could explain the variability in the effect of VDS [49]. Therefore, further studies should aim at evaluating not only the effect of vitamin D, but its activity in cancer risk reduction according to the patient’s receptors polymorphisms.

Vitamin D has also been proposed for COVID-19 cancer patients who were observed to have a higher risk of severe COVID-19 events. The immunomodulatory role of Vitamin D has long been known, and its antagonistic effect on viral replication in the respiratory tract, enteric infections, otitis media, Clostridium infections, vaginosis, urinary tract infections, sepsis, flu, dengue, hepatitis to be attributed to ability of vitamin D to increase antimicrobial peptides with antiviral and immunomodulatory activity [50]. A meta-analysis including 25 RCTs showed that supplementation with Vitamin D reduced by the incidence of acute respiratory infections two-thirds in subjects with levels of 25 (OH) D lower than 16 ng [51].

This is the first narrative review to simultaneously evaluate the effect of VDS in RCTs and observational studies, thus making it possible to obtain a comprehensive picture of all the existing scientific literature on the topic. Despite this, the present work is not without limitations, such as the limited number of papers eligible, the high degree of heterogeneity in several study characteristics in terms of vitamin D supplementation dose, concomitant therapies or supplementation, the length of treatments and follow-up, and the possibility of having missed some eligible studies. We did not evaluate the level of circulating 25(OH)D, because not all studies reported this information. Whether total 25(OH)D is the best indicator of vitamin D status is still a controversial issue, since there are actually divergent opinions for defining vitamin D status [52]. We were not able to take into account other factors, such as BMI, dietary intake, sun exposure, and all cancer sites and stages due to the limited data published in the original articles.

## 5. Conclusions

Evidence from RTCs and ObS studies does not presently make it possible to provide definitive answers on whether VDS has a beneficial impact on cancer survival/mortality. VDS has potential as treatment to improve survival in cancer patients, but none of the RCTs were conclusive due to limitations in the study designs. Only by critically reviewing the available evidence can we overcome those limitations to obtain a properly designed clinical trial. This evaluation could help researchers to plan well-designed and sufficiently powered RCTs to fully evaluate this association. Considering the uncertainty of the above results, we strongly recommend stratifying patients according to their baseline vitamin D status, preferring vitamin D3 supplementation, and diversifying the dose proposed according to the patients’ characteristics (e.g., BMI, cancer stage, patient’s receptor polymorphisms), as well as planning a proper duration of the supplementation and length of the follow-up according to outcomes.

## Figures and Tables

**Table 1:** Main characteristics of included studies (*n*. 25 articles: *n*. 8 RCT on cancer patients, *n.* 8 population RCT, *n*. 9 observational studies).

(a) Main Characteristics of RCTs in Cancer Patients							
First Author, Publication Year, Study Name	Cancer Site	Country	Participants	Sex	Age	Inclusion Criteria	Primary and Secondary Outcomes
Beer, 2007 [15] ASCENT	Prostate	US	250	Males	Range 45–92	Progressive metastatic androgen-independent prostate cancer—serum PSA 5.0 ng/mL, serum testosterone level 50 ng/dL, Eastern Cooperative Oncology Group performance status 2, life expectancy 3 months, age 18 yrs.	PSA reduction, OS, PSA, tumour, and clinical PFS, TRR measurable disease, skeletal morbidity-free survival, as well as safety and tolerability of the study treatment.
Attia, 2008 [16]	Prostate	US	70	Males	Range 52–85	>18 yrs of age, histologic diagnosis of prostate adenocarcinoma, radiographic evidence of metastasis, chemotherapy naive	PSA reduction, PFS, OS, ORR and toxicity
Scher, 2011 [17] ASCENT	Prostate	US	953	Males	70.9 VD and 70.4 control	Pathologically or cytologically proven adenocarcinoma of the prostate, metastatic disease and disease progression after medical or surgical castration (CRPC)	OS, thromboembolic event rates
Akiba, 2018 [18]	NSCLC	Japan	155	Males and females	68 (SD 9)	NSCLC (stage IA to IIIA), aged 20 to 75 yrs at entry; diagnosed and operated at one of four Jikei University Hospitals; tumour totally resected; no major complications; followed-up for as long as possible	RFS, OS
Golubic, 2018 [19]	CRC metastatic	Croatia	72	Males and females	69 (range 24–79)	CRC metastatic and 25(OH)D levels <75 nmol/L	OS, PFS
Ng, 2019 [20] SUNSHINE	CRC metastatic	US	139	Males and females	56 (Range 47–65)	Pathologically confirmed, unresectable locally advanced or metastatic CRC, no prior treatment, no previous VD supplementation	PFS, ORR, OS and change in plasma 25(OH)D level
Urashima, 2019 [21] AMATERASU	Digestive tract	Japan	417	Males and females	66 (Range 30–90)	Post-operative digestive tract cancer from the esophagus to the rectum, stages I to III, taking VD supplements or active VD; no history of urinary tract stones	RFS, OS, relapse, cancer-specific death, and no cancer death
Yonaga, 2019 [22] AMATERASU	Digestive tract	Japan	400	Males and females	Range 35–90	Post-operative digestive tract cancer from the esophagus to the rectum, stages I to III, taking VD supplements; no history of urinary tract stones	RFS, OS, relapse, cancer-specific death, and no cancer death
**(b) Main Characteristics of Population RCTs**							
**First Author, Publication Year, Study Name**	**Health Status**	**Country**	**Participants**	**Sex**	**Age**	**Inclusion Criteria**	**Primary and Secondary Outcomes**
Trivedi, 2003 [23]	General population	UK	2686	Males and females	VD group 74.8 (SD 4.6) and Placebo group 74.7 (SD 4.6) (range 65-85)	Fracture incidence	Fracture incidence and total mortality by cause.
Wactawski-Wende, 2006 [24] WHI	Postmenopausal women	US	36,282	Female	Range 50–79	Postmenopausal women 50 to 79 yrs enrolled in the WHI randomized trials	Prevent hip fracture, CRC
Chlebowski, 2008 [25] WHI	Postmenopausal women	US	36,282	Female	Range 50–79	Postmenopausal women 50 to 79 yrs enrolled in the WHI randomized trials	Hip fracture, breast and CRC
Brunner, 2011 [26] WHI	Postmenopausal women	US	36,282	Female	Range 50–79	Postmenopausal women 50 to 79 yrs enrolled in the WHI randomized trials	Prevent other fractures, or CRC
Avenell, 2012 [9] RECORD	Elderly	UK	5292	Males and females	77 (SD 6)	Fragility fracture within the last 10 yrs and aged at least 70 yrs	All-cause mortality, CVD mortality, cancer mortality, and cancer incidence
Ammann,2017 [27] WHI	Postmenopausal women	US	34,763	Female	Range 58-69	Postmenopausal women 50 to 79 yrs enrolled in the WHI randomized trials	Hip fracture, breast and CRC
Scragg, 2018 [11] VIDA	Community adults	NZ	5108	Males and females	65.9 (SD 8.3) (range 50–84)	50 to 84 yrs; resident of Auckland, New Zealand, at the time of recruitment; and anticipated residence in New Zealand for the 4-yrs study period	Cancer incidence and cancer mortality (Primary aim: assess the effect of VD suppl. on incidence of CVD)
Manson, 2019 [10] VITAL	General population	US	25,871	Males and females	67.1 (SD 7.1)	Men 50 yrs of age or older and women 55 yrs of age or older in the US	Cancer of any type and major CVE, site-specific cancers, cancer mortality, and additional CVE
**(c) Main Characteristics of Observational Studies**							
**First Author, Publication Year, Study Name**	**Health Status**	**Country**	**Participants**	**Sex**	**Age**	**Inclusion Criteria**	**Primary and Secondary Outcomes**
Poole, 2013 [35] (ABCPP)	Breast	US and China	12019	Female	58.0 (10.0) *	Breast cancer survivors, stage I–IV stage	BC recurrence, BC specific mortality, and all-cause mortality
Holm,2014 [28] (cohort study)	Breast	DK	1064	Female	62 (range 50–64)	Breast cancer diagnosis	BC specific mortality
Zeichner, 2015 [30](retrospective study)	Breast	US	246	Female	>50 years (users 53.0 SD 12.1)	VD with trastuzumab-based chemotherapy for HER2-positive (HER2D) nonmetastatic breast cancer	DFS, OS
Jeffreys, 2015 [33] (cancer register)	Breast, CRC, lung, ovarian or uterine	UK	21565	Female	>55 years	First diagnosis of breast, colorectal, lung, ovarian or uterine cancer in postmenopausal women identified at least 5 years of CPRD data prior to diagnosis and 3+ to 1–2 (but no more) VD prescriptions	Cancer survival
Wang, 2016 [32] (Longitudinal study)	Esophageal	China	303	Males and females	non-users 64.9 (SD 7.6) users 61.7 (SD 7.6)	Esophageal cancer patients undergoing esophagectomy post-surgery	QoL and survival
Lewis, 2016 [36] (cancer register)	CRC	US	453	Males and females	63.3 (SD 10.4)	Stage II CRC	VD supplementation and QoL
Mulpur, 2016 [31] (case-control)	Glioblastoma	US	470	Males and females	59 (median) (range 18-89)	>18 age, recent diagnosis of primary (nonrecurrent) GBM and undergoing treatment at participating medical and oncology centers in the South Eastern US	Associations of CAM use and GBM outcome/mortality
Madden, 2018 [35] (cancer register)	Breast	Ireland	5417	Female	non-users 68 (59–74) users 66 (59–73) (range 50–80)	Aged 50-80 yrs, stage I-II breast cancer diagnosis and no VD use in yr prior to diagnosis	BC specific mortality
Yokosawa, 2018 [29] (cohort study)	HNC	US	434	Males and females	NR	HNC diagnosis, >18 yrs	Death from any cause, HNC-specific death and recurrence of disease.

25(OH)D: 25 hydroxyvitamin D; ASCENT: Androgen Independent Prostate Cancer Study of Calcitriol Enhancing Taxotere; CAM: Complementary and Alternative Medicine; CPRD: Clinical Practice Research Datalink, UK; CRPC: Metastatic Castration-Resistant Prostate Cancer; CRC: Colon Rectal Cancer; CVE: Cardiovascular Event; CVD: Cardiovascular Disease; DFS: disease-free survival; GBM: Glioblastoma; GP: General Practice; HNC: Head and Neck Cancer; NSCLC: Non-Small-Cell Lung Cancer; ORR: Overall Response Rate; OS: Overall Survival; PFS: Progression-Free Survival; PSA: Prostate-Specific Antigen; QoL: Quality Of Life; RECORD: Randomized Evaluation of Calcium Or vitamin D; RFS: Relapse-Free Survival; TRR: Tumor Regression Rates; VD: Vitamin D; VIDA: the VItamin D Assessment; VITAL: The VITamin D and OmegA-3 TriaL; WHI: Women’s Health Initiative; Yrs: years; ABCPP: After Breast Cancer Pooling Project, consortium of four prospective cohorts. * age at diagnosis for users of single supplement (*n.* 5279).

**Table 2:** Details of the included studies (statistically significant estimates are in bold).

(a) Details of RCTs in Cancer Patients (Statistically Significant Estimates Are in Bold)								
First Author, Publication Year, Study Name	Arms	Intervention Dose/Day	Comparator	Duration of Treatment/ Follow-Up	Cancer Deaths/ Treatment	Cancer Deaths/ Controls	Contrast	Estimates
Beer, 2007 [15] ASCENT	2	45mcg DN-101 on day 1, 8 and 15 + therapy *	Placebo	Every 3 weeks/18.3 mths	NR	NR	VDS vs. placebo	OS HR = 0.67 (95% CI = 0.45–0.97)
Attia, 2008 [16]	2	10 mcg Doxercalciferol Orally/days 1–28 **	Placebo	Every 28 days/17.6 mths	31	25	VDS vs. placebo	OS Median 17.8 mths (95% CI = 14.9–23.6) vs. 16.4 mths (95% CI = 11.9-23.8) (P = 0.383)
Scher, 2011 [17] ASCENT	2	45mcg DN-101 on day 1, 8 and 15 + therapy *	Placebo	Every 3 weeks/11.7 mths	108	142	VDS vs. placebo	OS Median 17.8 mths (95% CI = 16.0–19.5) vs. 20.2 mths (95% CI = 18.8–23.0) (log-rank P = 0.002).
Akiba, 2018 [18]	2	1200 IU VD3/d	Placebo	12 months/3.3 yrs	40	24	VDS vs. placebo	OS HR = 1.22 (95% CI = 0.54–2.79)
Golubic, 2018 [19]	2	2000 IU/d + Standard chemotherapy	Placebo	2 years/46 mths	NR	NR	VDS vs. placebo	OS HR = 1.01 (95% CI = 0.39–2.61)
Ng, 2019 [20] SUNSHINE	2	8000 IU VD3/d followed by 4000 IU VD3/d ***	400 IU/d Standard dose	14 cycle/22.9 mths	45	54	High VDS vs. standard dose	OS Median 24.3 mths (95% CI = 19.0–33.2) vs. 24.3 mths (95% CI = 20.3–32.4) (log rank P = 0.43) OS HR = 0.64 (95% CI = 0–0.90)
Urashima, 2019 [21] AMATERASU	2	2000 IU VD/d	Placebo	3.5 yrs (median)	27	16	VDS vs. placebo	Cancer-specific death HR = 1.09 (95% CI = 0.58–2.01)
Yonaga, 2019 [22] AMATERASU	2	2000 IU VD/d	Placebo	3.5 years (median)	Well D AC: 19 Moderately D AC: 15 Poorly D AC: 3 Signet-ring CC: 1 SCC: 7	Well D AC: 13 Moderately D AC: 9 Poorly D AC: 8 Signet-ring CC: 4 SCC: 3	VDS vs. placebo	Well D AC OS HR = 0.82 (95%CI = 0.40–1.65) Moderately D AC OS HR = 1.31 (95%CI = 0.57–2.99) Poorly D AC OS HR = 0.25 (95%CI = 0.07–0.94) Signet-ring CC OS HR = 0.30 (95%CI = 0.03–2.65) SCC - OS HR = 1.39 (95%CI = 0.35–5.49)
**(b) Details of Population RCTs (In Bold Statistical Significant Estimates)**								
**First Author, Publication Year, Study Name**	**Arms**	**Intervention Dose/Day**	**Comparator**	**Duration of Treatment/** **Follow-Up**	**Cancer Deaths/** **Treatment**	**Cancer Deaths/** **Controls**	**Contrast**	**Estimates**
Trivedi, 2003 [23]	2	100 000 IU cholecalciferol	Placebo	Every 4 months for 5 yrs/5yrs	63	72	VDS vs. placebo	Cancer mortality RR = 0.86 (95% CI = 0.61–1.20)
Wactawski-Wende, 2006 [24] WHI	2	Calcium elemental 1000 mg + 400 IU VD3/d (two doses)	Placebo	7.0 ± 1.4 yrs	34	41	VDS vs. no VDS	CRC mortality HR = 0.82 (95% CI = 0.52–1.29)
Chlebowski, 2008 [25] WHI	2	Calcium elemental 1000 mg + 400 IU VD3/d (two doses)	Placebo	7 yrs	23	23	VDS vs. no VDS	Breast cancer mortality HR = 0.99 (95% CI = 0.55–1.76)
Brunner, 2011 [26] WHI	2	Calcium elemental 1000 mg + 400 IU VD3/d (two doses)	Placebo	7.0 ± 1.4 yrs	315	347	VDS vs. no VDS	Cancer mortality HR = 0.90 (95% CI = 0.77–1.05)
Avenell, 2012 [9] RECORD	4	800 IU VD3/day + 1000 mg Calcium/d	Placebo	3 yrs/ 6.2 yrs (median)	151	178	VDS vs. no VDS	Cancer mortality HR = 0.85 (95% CI= 0.68–1.06)
Ammann,2017 [27] WHI	2	Calcium elemental 1000 mg + 400 IU VD3/d (two doses)	Placebo	7 yrs	NR	NR	Calcium/VDS vs. placebo	Hematologic cancer-specific mortality HR = 0.77 (95% CI = 0.53–1.11)
Scragg, 2018 [11] VIDA	2	200,000 IU VD3 (initial bolus) followed by 100 000 IU/mths	Placebo	3 yrs/ 3.3 yrs (median)	44	45	NR	Cancer mortality HR = 0.97 (95% CI = 0.64–1.47)
Manson, 2019 [10] VITAL	2	2000 IU VD3 + ω3 = 1 g/d	Placebo	5 yrs/5.3 yrs (median)	154	187	NR	Cancer mortality HR = 0.83 (95% CI = 0.67–1.02)
**(c) Details of Observational Studies in Cancer Patients (In Bold Statistical Significant Estimates)**								
**First Author, Publication Year, Study Name**	**Arms**	**Intervention Dose/Day**	**Comparator**	**Duration of Treatment/** **Follow-Up**	**Cancer Deaths/** **Treatment**	**Cancer Deaths/** **Controls**	**Contrast**	**Estimates**
Poole, 2013 [35] (ABCPP)	2	Regular VD use at least 1-yr post diagnosis	No VDS	2.2 yrs (0.7) ^	41	808	Users vs. non-users	BC mortality HR = 0.97 (95% CI = 0.68–1.38)
Holm,2014 [28] (cohort study)	2	VD use previous 12 months: low mcg (200 IU)/d); medium (5–10 mcg (400 IU)/d); high (>10 mcg (400 IU)/d).	No VDS	6.3 yrs	60	45	for an increase in one category of the variable	BC mortality HR = 1.47 (95% CI = 1.07–2.00)
Zeichner, 2015 [30](retrospective study)	2	VD use during chemotherapy: <10,000 UI/week or >10,000 UI/week	No VD users	29.5 mths	NR	NR	Users vs. non-users	OS HR = 0.31 (95% CI = 0.11–0.89)
Jeffreys, 2015 [33] (cancer register)	2	Any suppl 5 yrs prior to cancer diagnosis	No VDS	30.4 mths	314	1789	Any vs. No suppl	BC survival HR = 0.78 (95% CI = 0.70–0.88)
					252	1474		CRC survival HR = 0.90 (95% CI = 0.78–1.04)
					443	2313		LC survival HR = 1.06 (95% CI = 0.96–1.17)
					134	1017		GC survival HR = 0.89 (95% CI = 0.73–1.07)
		≥3 prescriptions 5 yrs prior to cancer diagnois	1–2 prescriptions		228	86	≥3 prescriptions vs. 1–2 prescriptions	BC survival HR = 1.02 (95% CI = 0.79–1.32)
					191	61		CRC survival HR = 0.81 (95% CI = 0.59–1.11)
					323	120		LC survival HR = 0.86 (95% CI = 0.70–1.07)
					98	36		GC survival HR = 0.84 (95% CI = 0.59–1.30)
Wang, 2016 [32] (Longitudinal study)	2	Regular VD use after esophagectomy, during treatment and recovery phases: 200-400 IU/day	No VD users	24-mths after surgery	NR	NR	Users vs. non-users	OS HR = 0.80 (95% CI = 0.51–1.24)
Lewis, 2016 [36] (cancer register)	2	Regular VD use 12 months prior to cancer diagnosis	No VD users	24 months	NR	NR	Users vs. non-users	OS HR = 0.77 (95% CI = 0.37, 1.58)
Mulpur, 2016 [31] (case-control)	2	Regular VD use 5 yrs prior to cancer diagnosis	No VD users	1.3 wks to 5.3 yrs	373	NR	Users vs. non-users	OS HR = 0.72 (95% CI = 0.52–0.99)
Madden, 2018 [35] (cancer register)	2	De novo VD use post diagnosis	No VD users	NR	208	598	Users vs. non-users Users (initiation < 180d) vs. non-users Users (initiation ≥ 180d) vs. non-users	BC survival HR = 0.80 (95% CI = 0.64–0.99) BC survival HR = 0.51 (95% CI = 0.34–0.74) BC survival HR = 0.91 (95% CI = 0.70–1.18)
Yokosawa, 2018 [29] (cohort study)	3	Past use of VD: 0; 0-400 UI/day; ≥400 UI/day	Level of VDS	NR	32	28	Use of ≥400 UI vs. 0 UI	HNC survival HR = 1.11 (95% CI = 0.65–1.90)

25(OH)D: 25 hydroxyvitamin D; AC: Adenocarcinoma; ASCENT: Androgen Independent Prostate Cancer Study of Calcitriol Enhancing Taxotere; BC: Breast Cancer; CAM: Complementary and Alternative Medicine; CRC: Colon Rectum Cancer; D: differentiated; GC: Gynecologic Cancer; HNC: Head and Neck Cancer; LC: Lung Cancer; Mths: months; NR: not reported; ORR: Overall Response Rate; OS: Overall Survival; PFS: Progression-Free Survival; RECORD: Randomized Evaluation of Calcium Or vitamin D; SCC,: squamous cell carcinoma; Signet-ring: signet-ring cell carcinoma; suppl: supplementation; VD: Vitamin D; VDS: Vitamin D supplementation; VIDA: the VItamin D Assessment; VITAL: The VITamin D and OmegA-3 TriaL; WHI: Women’s Health Initiative; wks: weeks; yrs: years. HR = hazard ratio; RR = relative risk. * Weekly dose of 36 mg/m^2^ of docetaxel and 24 mg of dexamethasone for 3 weeks of a 4-week cycle. ** In addition to a 4-week cycle of docetaxel (35 mg/m^2^ i.v., days 1, 8, and 15), daily oral doxercalciferol were 10 mcg (initial), 7.5 mcg (dose level-1), 5.0 mcg (dose level-2), and 2.5 mcg (dose level-3). *** A continuous infusion of 2400 mg/m^2^ of 5-fluorouracil (5-FU) over 46 to 48 h, a bolus of 400 mg/m^2^ of 5-FU, 400 mg/m^2^ of leucovorin, and 85 mg/m^2^ of oxaliplatin (mFOLFOX6) plus 5 mg/kg of bevacizumab administered intravenously every 14 days (cycle). ^ years between diagnosis and study entry for users of single supplement (n. 5279).

## Data Availability

Not applicable.
